# MRI-Based Radiomics as a Promising Noninvasive Diagnostic Technique for Adenomyosis

**DOI:** 10.3390/jcm13082344

**Published:** 2024-04-18

**Authors:** Laurin Burla, Elisabeth Sartoretti, Manoj Mannil, Stefan Seidel, Thomas Sartoretti, Harald Krentel, Rudy Leon De Wilde, Patrick Imesch

**Affiliations:** 1 Department of Gynecology, University Hospital Zurich, 8091 Zurich, Switzerland; laurinburla@hotmail.com (L.B.);; 2 Department of Gynecology and Obstetrics, Hospital of Schaffhausen, 8208 Schaffhausen, Switzerland; 3 Faculty of Medicine, University of Zurich, 8032 Zurich, Switzerland; 4 Clinic for Radiology, Muenster University Hospital, 48149 Muenster, Germany; 5 Institute for Radiology and Nuclear Medicine, Hospital of Schaffhausen, 8208 Schaffhausen, Switzerland; 6 Department of Gynecology, Obstetrics and Gynecological Oncology, Bethesda Hospital Duisburg, 47053 Duisburg, Germany; 7 Clinic of Gynecology, Obstetrics and Gynecological Oncology, University Hospital for Gynecology, Pius-Hospital Oldenburg, Medical Campus University of Oldenburg, 26121 Oldenburg, Germany; 8 Clinic for Gynecology, Bethanien Clinic, 8044 Zurich, Switzerland

**Keywords:** adenomyosis, radiomics, MRI texture analysis

## Abstract

**Background:** MRI diagnostics are important for adenomyosis, especially in cases with inconclusive ultrasound. This study assessed the potential of MRI-based radiomics as a novel tool for differentiating between uteri with and without adenomyosis. **Methods:** This retrospective proof-of-principle single-center study included nine patients with and six patients without adenomyosis. All patients had preoperative T2w MR images and histological findings served as the reference standard. The uterus of each patient was segmented in 3D using dedicated software, and 884 radiomics features were extracted. After dimension reduction and feature selection, the diagnostic yield of individual and combined features implemented in the machine learning models were assessed by means of receiver operating characteristics analyses. **Results:** Eleven relevant radiomics features were identified. The diagnostic performance of individual features in differentiating adenomyosis from the control group was high, with areas under the curve (AUCs) ranging from 0.78 to 0.98. The performance of ML models incorporating several features was excellent, with AUC scores of 1 and an area under the precision-recall curve of 0.4. **Conclusions:** The set of radiomics features derived from routine T2w MRI enabled accurate differentiation of uteri with adenomyosis. Radiomics could enhance diagnosis and furthermore serve as an imaging biomarker to aid in personalizing therapies and monitoring treatment responses.

## 1. Introduction

In adenomyosis, endometrial-type glandular tissue and stroma are located in the myometrium [1,2,3]. The condition is associated with infertility, pelvic pain, menorrhagia, dysmenorrhea, and obstetrical complications [4,5,6]. The quality of life of affected women and the health care burden are relevant, as 37.6% use chronic pain medications and 82.0% undergo hysterectomy [7]. Medical and surgical treatment options can reduce adenomyosis-related symptoms, but there is currently no evidenced-based approach—especially for infertile patients with adenomyosis [8,9]. Transvaginal ultrasound (TVUS) is the diagnostic tool of choice and has a sensitivity of 74% and a specificity of 76% (two-dimensional TVUS). In recent years, the MUSA working group has developed criteria to enable standardized diagnostics [10,11]. 

MRI plays a role in treatment planning for complex cases where ultrasound is inconclusive, especially in combination with deep endometriosis [12,13,14]. MRI is reported to have a sensitivity of 78% and a specificity of 88% for diagnosing adenomyosis [15]. There are various MRI signs for adenomyosis, such as the junctional zone (JZ) thickness, an irregular appearance of the JZ, uterine morphology, and focal thickening [13,15]. To our knowledge, there are no internationally recognized guidelines for MRI diagnostics in adenomyosis, and with the mentioned criteria, the potential of this imaging modality does not seem fully realized, especially when looking beyond its scope into other medical fields [12,16]. 

In the field of medical imaging, radiomics has gained traction as a means of enhancing the diagnostic yield and predictive power of medical images. With radiomics, high-dimensional mineable data that cannot be detected by the human eye are extracted from medical images and leveraged as biomarkers [17,18]. The first data on radiomics in adenomyosis have been published, but to our knowledge, there has been no study addressing its diagnostic utility [19]. We hypothesized that radiomic features derived from T2w MRI could enable the accurate, noninvasive, and reliable MRI-based diagnosis of adenomyosis.

## 2. Materials and Methods

### 2.1. Patient Selection

In this retrospective observational single-center proof-of-principle study, consecutive female patients from the Endometriosis Registry of Hospital Schaffhausen, Switzerland, were examined. The recruitment period spanned from 1 January 2013 to 31 December 2021. The test group included patients with histologically confirmed adenomyosis, who were compared to a control group without histological evidence of adenomyosis. Only premenopausal patients were included in both groups. Patients were included in the adenomyosis group based on the following criteria: clinical suspicion of adenomyosis derived from medical history and clinical examination, MRI-based preoperative imaging findings, and availability of histology after hysterectomy as the reference standard. Exclusion criteria encompassed the absence of adenomyosis in histopathology or faulty MRI sequences unsuitable for evaluation. Patients were included in the control group if they had MRI-based preoperative imaging findings and availability of histology after hysterectomy as the reference standard. They were excluded if adenomyosis was present or if faulty MRI sequences were identified.

The aim of this study was to investigate the diagnostic potential of MRI-based radiomics as a novel, non-invasive diagnostic tool for adenomyosis. By utilizing radiomics features in routine T2w MRI scans, which represent the gold standard for endometriosis and adenomyosis, this study seeked to enable accurate differentiation between uteri with and without adenomyosis [20].

The histopathological evaluation was conducted by the Institute of Pathology, Hospital of Winterthur, Switzerland. The histopathological assessment followed these criteria: Adenomyosis is defined as a lesion where endometrial stroma and glands are located within the myometrium. Since the lower boundary of the endometrium toward the myometrium is typically irregular, adenomyosis is diagnosed only if the lesions were clearly below this line, typically at a distance of at least 2 mm.

In cases of suspected clinical diagnosis, either macroscopically evident areas are embedded in addition to the routine block of the endomyometrium, or in the absence of macroscopic abnormalities, 2–3 additional arbitrary paraffin blocks are taken from the corpus. However, there are no universally accepted guidelines in this regard.

### 2.2. MRI

**Figure 1:**
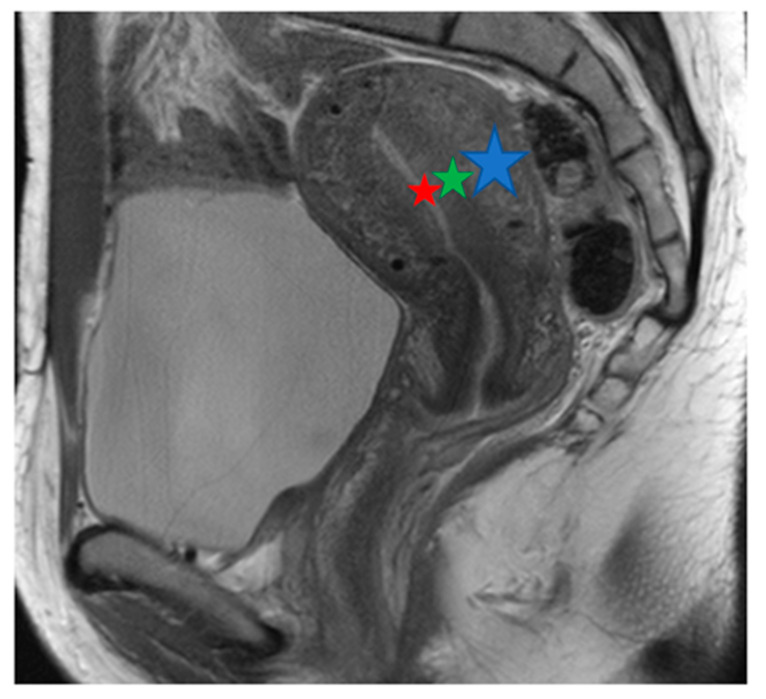
Normal uterus: T2w sagittal image. MRI displays the zonal anatomy of the uterus. The endometrium has a high T2 signal (red star); the inner myometrium has a low T2 signal, known as the junctional zone (green star). The outer myometrium has an intermediate signal on T2 (blue star).

**Figure 2:**
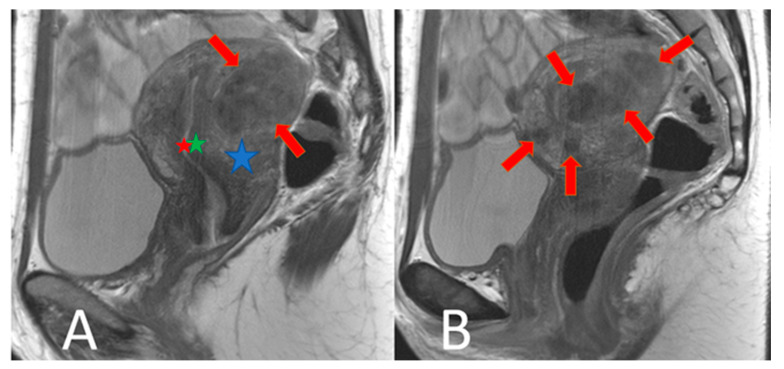
Focal adenomyosis: T2w sagittal images. (**A**): The uterus is enlarged by multiple T2 non-homogeneously hypointense nodules of different sizes in the outer myometrium (red arrows). The endometrium (red star) and the inner myometrium (green star) are outlined. The outer myometrium (blue star) is partly visible. (**B**) shows another analogous example.

**Figure 3:**
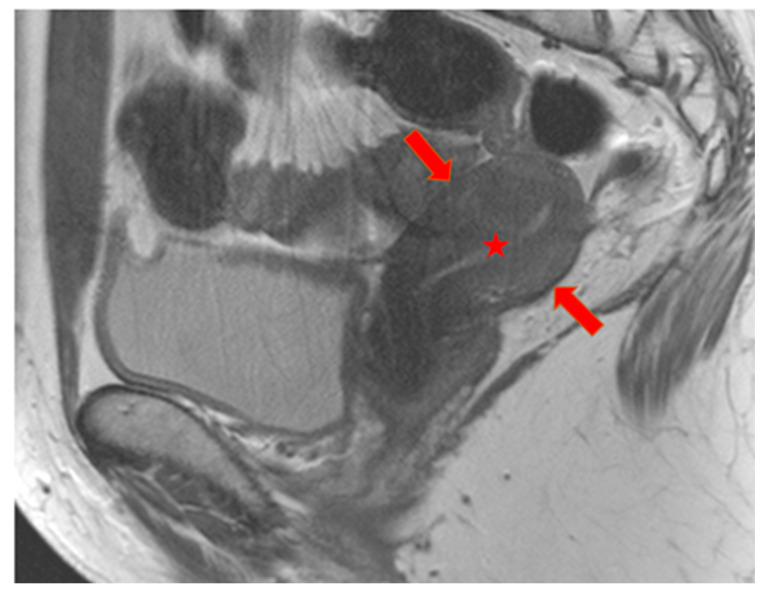
Diffuse adenomyosis: T2w sagittal image. The uterus is diffusely and homogeneously hypointense without differentiation of the inner and outer myometrium (red arrows). A T2 hyperintense myometrium (red star) is visible.

Prior to hysterectomy, all patients underwent a routine and comprehensive pelvic MRI examination on the same standard clinical 3T scanner (Achieva; Philips Healthcare, Best, The Netherlands). For further analyses, only sagittal T2w images were considered with the following parameters: 3D T2w Turbo Spin Echo (TSE) sequence, Cartesian acquisition, repetition time (TR) of 4279 ms, echo time (TE) of 70 ms, flip angle of 90°, field of view (FOV) of 370 × 349.4 × 180 mm^3^, acquired voxel size of 0.47 mm, reconstructed voxel size of 0.36 mm, 60 slices, 2.0 signal averages (NSAs), and a total scan duration of 07 min and 42 s. Figure 1 shows a normal uterus, Figure 2 shows a uterus with predominantly focal adenomyosis, and Figure 3 shows a uterus with diffuse adenomyosis. 

### 2.3. Image Segmentation and Radiomic Feature Extraction

Image segmentation was performed using the opensource software platform 3D Slicer (version 4.8, www.slicer.org) URL (accessed on 1 December 2021). After loading the Digital Imaging and Communications in Medicine (DICOM) files into the program (sagittal T2w images), segmentation was performed manually by outlining the uterus in all slices. Before feature extraction, all segmented images underwent standardized preprocessing as follows: spatial resampling to 1 × 1 × 1 mm^3^ using a fifth-degree LaGrangian polynomial, intensity discretization to a bin width of 25, and relative intensity rescaling using a scale of 500 [21]. Then, a total of 884 features were extracted per patient using the pyRadiomics package, which was implemented as a plugin in the 3D Slicer platform. 

Radiomics features corresponded to 7 different matrices/feature classes: a first-order statistics/histogram matrix, shape-based features, a gray-level co-occurrence matrix (GLCM), a gray-level run length matrix (GLRLM), a gray-level size zone matrix (GLSZM), a neighboring gray tone difference matrix (NGTDM), and a gray-level dependence matrix (GLDM). Additionally, images underwent a wavelet transformation. From these images, wavelet-based features were extracted, which contained all first-order statistics features and textural features, but were extracted from images with this wavelet decomposition [22]. A detailed description of all features can be found in the pyRadiomics documentation and elsewhere [23].

### 2.4. Dimension Reduction and Feature Selection

All features were first subjected to a normalization procedure using Z-score standardization [24]. Then, features were used as input data for the Boruta dimension-reduction and feature-elimination algorithm. The Boruta algorithm compares the importance of the real predictor features with those of random features (called shadow features) by means of statistical testing and several runs of the random forest (RF) method. Randomness is added to a prescribed dataset by building all the features of a mixed copy (shadow features). 

Next, training is carried out with an extended dataset of RF classifications, and a feature importance measure is implemented (the default setting is the average reduction accuracy). A higher importance score indicates the greater importance of the feature. In each iteration, the algorithm checks whether a real feature is more important than the best shadow feature (specifically, whether the feature receives higher scores than the largest shadow feature). If the real feature does not score as high as the shadow feature, it is no longer considered a very important feature. The process is finally terminated when all features are detected or when the algorithm achieves a given limitation for the RF operation [25]. 

### 2.5. Data Analysis

All statistical analyses were performed in R (version 4.02; R Foundation for Statistical Computing) using the packages “Boruta” and “healthcare.ai”. Features selected for further analyses were first subjected individually to receiver operating curve (ROC) analyses. To this end, the area under the curve (AUC) and its 95% confidence interval (CI) were computed. Two models were fitted to quantify the overall diagnostic performance of the combined metrics in differentiating adenomyosis cases from the control group. 

In the first model, all remaining features were implemented as predictor variables. In the second model, only a subset of the remaining features were implemented as predictor variables. Specifically, only non- to less correlated features were implemented in the second model. The Boruta algorithm does not account for collinearity in the data. Thus, from clusters of highly correlated features detected in a correlation matrix, only select features from each cluster should be used. 

**Figure 4:**
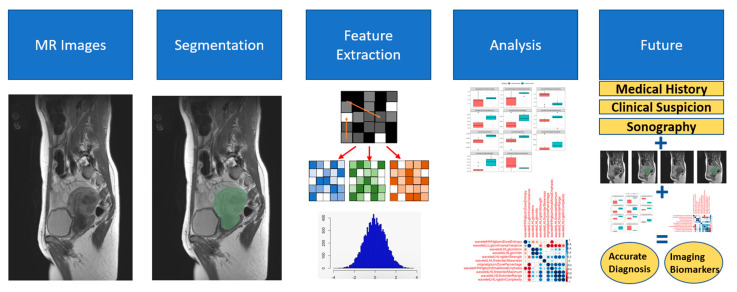
Schematic representation of segmentation, feature extraction, analysis, and future potential of radiomics. This figure illustrates the process of how the radiomics features were derived from T2w MRI and especially illustrates how the image segmentation and feature extraction were performed. Additionally, the future potential of using radiomics in combination with clinical data is highlighted.

For each model, three algorithms were individually fitted and optimized iteratively: random forest, extreme gradient boosting, and regularized regression. The models were tuned via 5-fold cross validation with 10 combinations of hyperparameter values. The optimal algorithm with the optimal hyperparameter values for a given model was then selected based on the AUC-ROC performance metric. Due to the very limited sample size, we did not reserve a subset of the data as a validation set. Thus, the results are based solely on model training. Figure 4 illustrates the process of segmentation, feature extraction, and analysis and provides an idea of the future of radiomics. 

## 3. Results

### 3.1. Patients

After excluding two patients due to faulty MRI sequences, the study included 15 patients for definitive analysis. Nine patients (40.8 ± 4.7 years old (mean ± SD)) had received a diagnosis of adenomyosis based on histopathological analysis, while in six patients (40.3 ± 3.9 years old), histopathological examination revealed no signs of adenomyosis. A third (66.6%; *n* = 6) of patients in the adenomyosis group (average duration of therapy 7.7 months) and 50% (*n* = 3) of the control group (average duration 3 months) underwent endocrine therapy directly before the MRI. The MRI of one person in the adenomyosis group and of one person in the control group showed multiple myomas. Intraoperatively, endometriosis was found in all patients (Table 1). 

### 3.2. Radiomics

**Figure 5:**
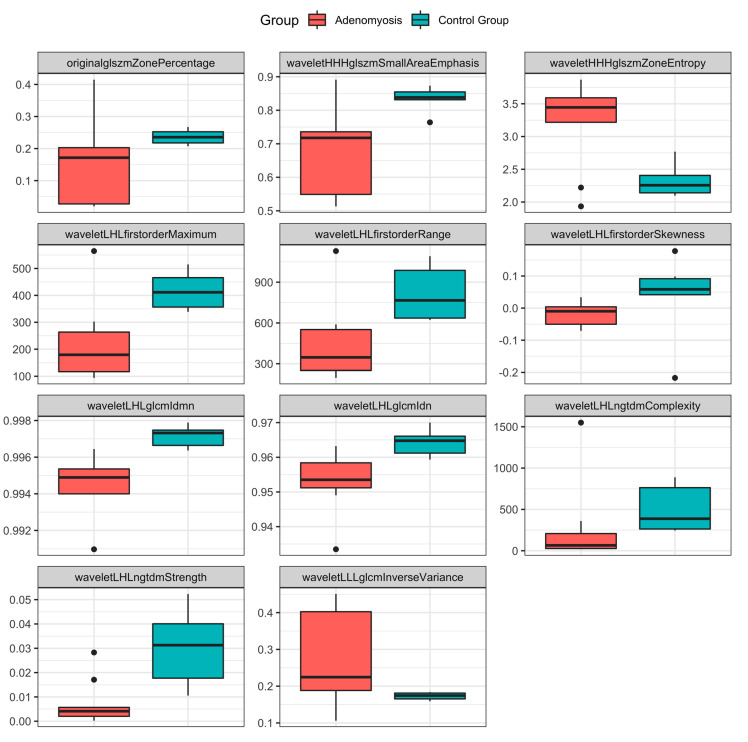
Boxplots of radiomics features stratified by patient group. Boxplots showing the distribution of values for the 11 features, stratified by patient group. The lines in the boxes show the median, the lower and upper hinges correspond to the first and third quartiles, the upper/lower whiskers extend from the hinges to the largest/smallest values no further than 1.5 * interquartile range from the hinges. The black dots represent outliers.

**Figure 6:**
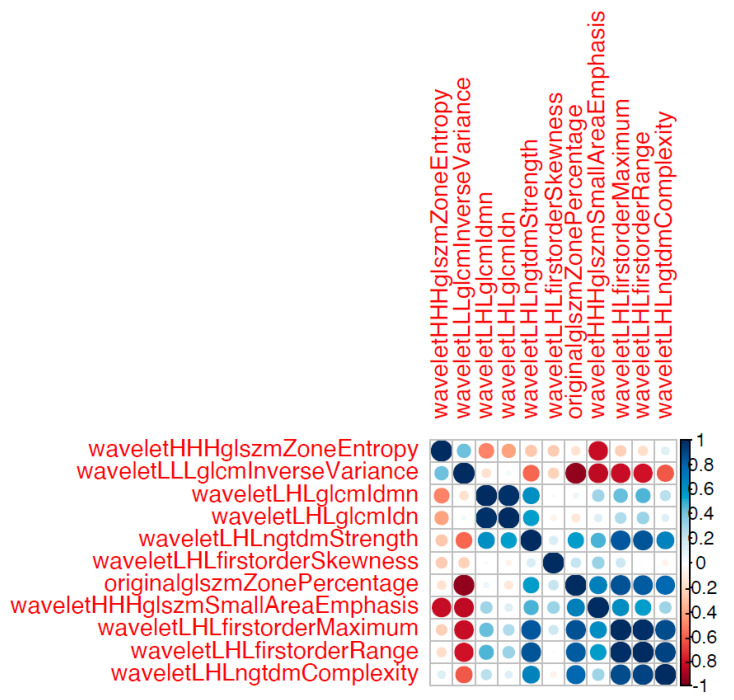
Correlation matrix of all 11 remaining features. The color and size of the circles is proportional to the magnitude of the correlation between two given features (Pearson correlation coefficients) as shown in the legend on the right side of the figure. Five features stemming from clusters of highly correlated features were selected for model 2.

After dimension reduction and feature selection, 11 features remained (Table 2). A detailed description of these features can be found in the literature [22,23]. Figure 5 provides a visual representation of the diagnostic capabilities of these features. In brief, the AUC of these features ranged from 0.78 to 0.98, indicating the high diagnostic performance of the individual features in differentiating patients with adenomyosis from patients in the control group. As for the performance of the combined metrics, Model 1 (encompassing all 11 features) achieved an excellent AUC-ROC score of 1 and an area under the precision-recall (AUPR) curve score of 0.4. Model 2 (encompassing only five non- to less correlated features, Figure 6) also achieved an excellent AUC-ROC score of 1 and an AUPR score of 0.4 (Figure 6).

## 4. Discussion

This proof-of-principle study provides what appears to be the first assessment of the utility of MRI-based radiomics for the diagnosis of adenomyosis. Our results indicate that the diagnostic yield of certain radiomics features is high and could potentially allow for a more accurate and noninvasive diagnosis of adenomyosis. With the development of in-depth image analysis tools and machine learning (ML) methods, the field of radiomics has emerged as a promising approach to expand the diagnostic yield of medical images. Radiomics is an established technique that is already being used in various medical fields, including gynecology [26,27,28,29,30].

We identified 11 radiomics features from T2w MRI that have high diagnostic performance in detecting adenomyosis. The AUC scores of individual features ranged from 0.78 to 0.98, indicating a high to very high diagnostic performance. Diagnostic performance was further improved when implementing several ML-backed models incorporating various features to quantify the overall performance of the radiomics features. Importantly, 10 of the 11 features were wavelet-based.

MRI is generally considered a reliable tool for diagnosing adenomyosis, and the standard diagnostic sequence is the non-contrast-enhanced T2w spin-echo sequence, as used for endometriosis [20]. Currently, the most important (conventional) imaging features that are used to diagnose adenomyosis are the characteristics of the JZ. For example, thickening of the JZ exceeding 12 mm is widely recognized as a marker of adenomyosis, but recent studies suggest that its diagnostic value is limited. Other features commonly used in everyday assessment include uterine morphology, irregular JZ, signal intensity in myometrial foci, and the presence of myometrial cysts [13,15,31]. However, the conventional MRI has its limitations. The lack of standardized definitions and clear boundaries for the junctional zone complicates diagnosis. While MRI signals show strong contrasts, histopathological changes are subtle. Direct indicators such as subendometrial lines on TVUS or bright foci in MRI are more dependable markers of adenomyosis compared to solely relying on thickened junctional zones [32]. The radiological assessment of adenomyosis is not well standardized, and internationally recognized guidelines are lacking [16].

Radiomics could be helpful, even more with the simultaneous presence of benign uterine diseases such as fibroids, which negatively impact the accuracy of TVUS and MRI [5,11]. Improving the diagnosis and, consequently, the treatment of adenomyosis is thus of paramount importance, as improved diagnostic capabilities could help minimize over- and underdiagnosis of the condition [33]. Radiomics could contribute to making the diagnosis safer than that using conventional MRI and TVUS, thus helping to minimize over and underdiagnosis.

With radiomics, high-dimensional mineable data that cannot be detected by the human eye can be extracted from medical images. These radiomics features can be leveraged as clinically relevant biomarkers and can thus enhance the predictive and diagnostic capabilities of medical images. Exemplarily, radiomics may be used to detect whether a disease is present or not, as in the current study [19,34]. Through the more differentiated diagnosis and localization of adenomyosis in the myometrium, more individualized therapy could be facilitated.

Radiomics could also be employed as a follow-up modality to track endocrine therapies and their treatment responses, which has already been explored in part with conventional MRI [35]. Furthermore, it could also be used to monitor the outcomes of interventional therapies. Surgical procedures and treatments like high-intensity focused ultrasound could be more precisely planned, and radiomics could enable monitoring of disease progression and response to therapies [19,30].

With regards to adenomyosis, radiomics could open up a new research field and contribute to better understanding of the disease itself by providing in-depth insights into imaging data. This could provide insights into the pathomechanisms of adenomyosis. It might also prove beneficial in the context of endometriosis. Radiomics could help improve the understanding of different disease manifestations as an imaging biomarker. Additionally, insights into the pathomechanisms of diseases could be gained by analyzing synchronous occurrences of deep endometriosis and adenomyosis.

Initial studies have explored the potential of artificial intelligence methods such as machine and deep learning in gynecological imaging overall, as well as in endometriosis and adenomyosis, and the findings appear promising [36,37,38,39,40]. For instance, in a sonographic preliminary study, a deep learning model showed a lower accuracy but a higher specificity in diagnosing adenomyosis on ultrasonographic images compared to intermediate-skilled trainees [36]. These techniques are already advanced and have been further investigated in other medical fields, often by radiologists. The lag in the field of gynecology, particularly in endometriosis and adenomyosis, may be attributed to imaging being predominantly handled by gynecologists in this area, who may be less familiar with these techniques. However, these techniques are rapidly emerging, not only in medicine but also in other fields, underscoring the importance of gynecological specialties embracing these advancements early on [41].

The age of the premenopausal patients in this study was comparable between both groups, but averaged at around 40 years, which is relatively high (Table 1). This is primarily due to the fact that the patients in this study were intended to undergo hysterectomy. However, obtaining further insights into a younger patient population would certainly be crucial. Diagnosis in adolescent patients poses a challenge, often with long latency periods before diagnosis and treatment initiation [42,43]. Radiomics could have potential in supporting early diagnosis in this context.

Some of the patients underwent endocrine therapy prior to the MRI scan. This could potentially influence the Radiomics analysis, as could the interval between the MRI and surgery (Table 1). However, the latter is relatively short, typically only a few months, so a significant impact is not expected in this study. Future studies with larger populations should focus on assessing disease activity and progression using radiomics, as well as examining the influence of endocrine therapy.

Special consideration should also be given towards the costs associated with the use of radiomics. The integration of radiomics into clinical practice may lead to additional expenses associated with the implementation of specialized software, computational resources, and training for personnel involved in image analysis. However, it is important to recognize that these initial costs may be offset by potential long-term benefits such as improved diagnostic accuracy, personalized treatment planning, and enhanced patient outcomes. Additionally, the utilization of radiomics has the potential to streamline workflows, reduce the need for invasive procedures, and minimize unnecessary imaging studies, thereby contributing to overall cost-effectiveness in healthcare delivery.

Furthermore, the impact of radiomics on the length of time for executing exams is a significant consideration. While the extraction and analysis of radiomic features may introduce additional postprocessing time, advancements in computational techniques and automation have the potential to mitigate this concern. Automated feature extraction algorithms and machine learning approaches can expedite the analysis process, thereby minimizing delays in the interpretation of medical images. Moreover, the integration of radiomics into existing imaging protocols may facilitate more efficient data acquisition and interpretation workflows, ultimately reducing the overall time required to perform imaging exams. With ongoing technological advancements and the increasing accessibility of imaging resources, the integration of radiomics into clinical practice has the potential to be applicable in a wide range of contexts, including community hospitals, outpatient clinics, and remote healthcare facilities.

Our study has several limitations: Despite the potential of these 11 radiomics features, it should be acknowledged that the sample size was limited. Thus, it is possible that other feature classes may be identified as relevant and clinically significant in future studies. The identification of relevant features depends on the patient population and other factors, such as different MRI acquisition parameters. Radiomics features should be considered as image-based biomarkers that are subject to various confounding factors [44]. Therefore, the value of this work is not fundamentally in the unambiguous identification of the relevant features, but rather in the proof that adenomyosis leaves a unique and strong MRI-based biosignature that can be leveraged for the noninvasive detection of adenomyosis.

The sample size is limited for several reasons: it is a single-center study and a proof-of-principle investigation. To our knowledge, this specific topic has never been explored and published in this manner before; hence, our initial aim was to start with a smaller population. Additionally, all patients were required to undergo an MRI, which is not standard practice, as well as a hysterectomy for histological reference.

Despite the limited sample size, we applied ML to our dataset to quantify the overall diagnostic performance of the combined radiomics features. However, overfitting is a concern. We tried to counteract this by implementing cross validation. Additionally, our study was performed using a single MRI scanner, which limits the generalizability of our findings. The influence of different acquisition parameters, sequences, field strengths, and MRI scanners concerning the reliability of radiomics features remains to be investigated [45]. Lastly, our results depend on the quality of the segmentation. To counteract this, all uteri were segmented twice to decrease reader-dependent bias.

This is a proof-of-principle study and not a conclusive work. Our objective was to provide an impetus for future studies and demonstrate that radiomics in adenomyosis is a promising and practically overlooked field, despite numerous studies in other medical domains. Larger, multicenter studies are necessary based on our proof-of-principle study. Future research projects should investigate the results presented here in a broader population and compare the diagnostic value of radiomics for different forms of adenomyosis with conventional MRI and TVUS. Additionally, exploring Radiomics as an imaging biomarker is warranted. It would be interesting to address questions regarding the pathomechanism of adenomyosis, different disease phenotypes, their differentiation, and similarities with deep endometriosis when occurring simultaneously. Furthermore, tracking symptomatology and disease progression using radiomics would be compelling. Do endocrine therapies influence the analysis? Could radiomics lead to more personalized therapies by providing better insights into treatment responses, disease activity, and progression, especially for women with a desire to conceive? How beneficial would radiomics be as a follow-up modality after various therapies?

In conclusion, with this proof-of-principle study, we have demonstrated for the first time, to the best of our knowledge, that radiomics features can enable a precise non-invasive MRI-based diagnosis of adenomyosis. Radiomics represents a promising diagnostic modality and could open up a new, interesting field of research for adenomyosis. Improving the non-invasive diagnosis of adenomyosis is crucial for gaining a better understanding of the disease. As seen in other medical areas, radiomics not only shows the potential to determine the presence of a condition but could also serve as a diagnostic biomarker to better understand pathomechanisms, support therapy individualization, and act as a follow-up modality for various treatments.

## Figures and Tables

**Table 1:** Patient details. In this table, the patient details of the two groups are presented (AG: Adenomyosis Group, histologically confirmed adenomyosis; CG: Control Group, no histological evidence of adenomyosis). The patients are numbered within each group. Age at MRI is provided (in years). G/P (Gravida/Para). The endocrine therapy as follows in the table (0: none, 1: COC (combined oral contraceptive), 2: Gestagen (Dienogest), 3: Levonorgestrel-IUD, 4: GnRH-Analogue). In the context of endocrine therapy and duration, therapies taken prior to the MRI are referred to. Additionally, preoperative TVUS based on MUSA criteria, conventional MRI, and intraoperative endometriosis findings based on #Enzian (s) are shown.

Patients	Age	G/P	Endocrine Therapy	Endocrine Therapy Duration before MRI (Months)	Adenomyosis in TVUS	Adenomyosis in Conventional MRI	Duration MRI—Surgery (Months)	Endometriosis (Intraoperative #Enzian)
AG								
1	33	0/0	2	1	No	Yes	4	P2, T3/3, A2, B2/1, C1, FA
2	42	0/0	1	36	Yes	Yes	5	P1, FA
3	44	II/I	0	0	Yes	Yes	3	P1, O1/1, T1/1, A3, B2/1, FA, FB
4	35	I/I	2	2	Yes	No	1	P2, O1/1, T1,1, A2, FA, FB
5	47	II/II	3	3	Yes	Yes	1	P2, O2/2, T3/3, A2, B1/1, C1, FA, FB
6	42	0/0	4	2	Yes	Yes	5	P2, O1/0, T3/1, B1/0, FA
7	41	I/I	0	0	Yes	Yes	2	P2, O1/1, T1/1, A3, B1/1, C3, FA, FB
8	39	0/0	0	0	Yes	Yes	1	P3, T2/0, A3, B2/1, C1, FA, FU
9	44	III/II	2	2	Yes	Yes	1	P3, O3/3, T3/3, A3, B3/3, C2, FA, FU, FI (Sigma)
CG								
1	38	II/I	0	0	No	No	2	P1, O1/1, T0/1, B1/1, FB
2	48	I/I	2	1	No	Yes	1	P2, O2/0, B1/1
3	37	IV/III	2	3	No	Yes	1	P1, O2/1, T1/1, A1
4	40	II/II	3	5	No	No	2	P1, O1/2, T1/1, A2, B1/0, C1
5	39	II/II	0	0	Yes	Yes	4	FB
6	40	0/0	0	0	No	Yes	2	FO (umbilicus)

**Table 2:** Radiomics features.

Feature	AUC (95% Confidence Interval)
Original_glszm_ZonePercentage	0.78 (0.49–1)
Wavelet_LHL_glcm_Idmn	0.98 (0.93–1)
Wavelet_LHL_glcm_Idn	0.93 (0.79–1)
Wavelet_LHL_firstorder_Skewness	0.83 (0.51–1)
Wavelet_LHL_firstorder_Maximum	0.89 (0.67–1)
Wavelet_LHL_firstorder_Range	0.89 (0.67–1)
Wavelet_LHL_ngtdm_Complexity	0.83 (0.6–1)
Wavelet_LHL_ngtdm_Strength	0.91 (0.76–1)
Wavelet_HHH_glszm_SmallAreaEmphasis	0.83 (0.6–1)
Wavelet_HHH_glszm_ZoneEntropy	0.83 (0.6–1)
Wavelet_LLL_glcm_InverseVariance	0.78 (0.49–1)

This table describes the 11 features remaining after dimension reduction and feature selection.

## Data Availability

The datasets analyzed during the current study are available from the corresponding author upon reasonable request.

## Abbreviations

MRI	Magnetic Resonance Imaging
T2w	T2-weighted
SD	Standard Deviation
TVUS	Transvaginal Ultrasound
TSE	Turbo Spin Echo
TR	Repetition Time
TE	Echo Time
FOV	Field of View
NSA	Number of Signal Averages
ML	Machine Learning
ROC	Receiver Operating Curves
AUC	Area under Curve
AUPR	Area under the Precision- Recall curve
DICOM	Digital Imaging and Communications in Medicine
GLCM	Gray-level Co-occurrence Matrix
GLRLM	Gray-level Run Length Matrix
GLSZM	Gray-level Size Zone Matrix
NGTDM	Neighboring Gray Tone Difference Matrix
GLDM	Gray-level Dependence Matrix
RF	Random Forest
CI	Confidence Interval
JZ	Junctional Zone

## References

[1] Vannuccini S., Tosti C., Carmona F., Huang S.J., Chapron C., Guo S.W., Petraglia F. (2017). Pathogenesis of adenomyosis: An update on molecular mechanisms. Reprod. Biomed. Online.

[2] Loring M., Chen T.Y., Isaacson K.B. (2021). A Systematic Review of Adenomyosis: It Is Time to Reassess What We Thought We Knew about the Disease. J. Minim. Invasive Gynecol..

[3] Guo S.W. (2020). The Pathogenesis of Adenomyosis vis-à-vis Endometriosis. J. Clin. Med..

[4] Horton J., Sterrenburg M., Lane S., Maheshwari A., Li T.C., Cheong Y. (2019). Reproductive, obstetric, and perinatal outcomes of women with adenomyosis and endometriosis: A systematic review and meta-analysis. Hum. Reprod. Update.

[5] Garcia L., Isaacson K. (2011). Adenomyosis: Review of the literature. J. Minim. Invasive Gynecol..

[6] Komatsu H., Taniguchi F., Harada T. (2023). Impact of adenomyosis on perinatal outcomes: A large cohort study (JSOG database). BMC Pregnancy Childbirth.

[7] Yu O., Schulze-Rath R., Grafton J., Hansen K., Scholes D., Reed S.D. (2020). Adenomyosis incidence, prevalence and treatment: United States population-based study 2006–2015. Am. J. Obstet. Gynecol..

[8] Harada T., Taniguchi F., Guo S.W., Choi Y.M., Biberoglu K.O., Tsai S.S., Alborzi S., Al-Jefout M., Chalermchokcharoenkit A., Sison-Aguilar A.G. (2023). The Asian Society of Endometriosis and Adenomyosis guidelines for managing adenomyosis. Reprod. Med. Biol..

[9] Dason E.S., Maxim M., Sanders A., Papillon-Smith J., Ng D., Chan C., Sobel M. (2023). Guideline No. 437: Diagnosis and Management of Adenomyosis. J. Obstet. Gynaecol. Can..

[10] Harmsen M.J., Van den Bosch T., de Leeuw R.A., Dueholm M., Exacoustos C., Valentin L., Hehenkamp W.J.K., Groenman F., De Bruyn C., Rasmussen C. (2022). Consensus on revised definitions of Morphological Uterus Sonographic Assessment (MUSA) features of adenomyosis: Results of modified Delphi procedure. Ultrasound Obstet. Gynecol..

[11] Krentel H., Keckstein J., Füger T., Hornung D., Theben J., Salehin D., Buchweitz O., Mueller A., Schäfer S.D., Sillem M. (2023). Accuracy of ultrasound signs on two-dimensional transvaginal ultrasound in prediction of adenomyosis: Prospective multicenter study. Ultrasound Obstet. Gynecol..

[12] Chapron C., Vannuccini S., Santulli P., Abrão M.S., Carmona F., Fraser I.S., Gordts S., Guo S.W., Just P.A., Noël J.C. (2020). Diagnosing adenomyosis: An integrated clinical and imaging approach. Hum. Reprod. Update.

[13] Rees C.O., Nederend J., Mischi M., van Vliet H.A.A.M., Schoot B.C. (2021). Objective measures of adenomyosis on MRI and their diagnostic accuracy—A systematic review & meta-analysis. Acta Obstet. Gynecol. Scand..

[14] Bazot M., Daraï E. (2018). Role of transvaginal sonography and magnetic resonance imaging in the diagnosis of uterine adenomyosis. Fertil. Steril..

[15] Tellum T., Nygaard S., Lieng M. (2020). Noninvasive Diagnosis of Adenomyosis: A Structured Review and Meta-analysis of Diagnostic Accuracy in Imaging. J. Minim. Invasive Gynecol..

[16] Zhang M., Bazot M., Tsatoumas M., Munro M.G., Reinhold C. (2023). MRI of Adenomyosis: Where Are We Today?. Can. Assoc. Radiol. J..

[17] Aerts H.J., Velazquez E.R., Leijenaar R.T., Parmar C., Grossmann P., Carvalho S., Bussink J., Monshouwer R., Haibe-Kains B., Rietveld D. (2014). Decoding tumour phenotype by noninvasive imaging using a quantitative radiomics approach. Nat. Commun..

[18] Lambin P., Leijenaar R.T.H., Deist T.M., Peerlings J., de Jong E.E.C., van Timmeren J., Sanduleanu S., Larue R.T.H.M., Even A.J.G., Jochems A. (2017). Radiomics: The bridge between medical imaging and personalized medicine. Nat. Rev. Clin. Oncol..

[19] Li Z., Zhang J., Song Y., Yin X., Chen A., Tang N., Prince M.R., Yang G., Wang H. (2021). Utilization of radiomics to predict long-term outcome of magnetic resonance-guided focused ultrasound ablation therapy in adenomyosis. Eur. Radiol..

[20] Bazot M., Bharwani N., Huchon C., Kinkel K., Cunha T.M., Guerra A., Manganaro L., Buñesch L., Kido A., Togashi K. (2017). European society of urogenital radiology (ESUR) guidelines: MR imaging of pelvic endometriosis. Eur. Radiol..

[21] Baessler B., Nestler T., Pinto Dos Santos D., Paffenholz P., Zeuch V., Pfister D., Maintz D., Heidenreich A. (2020). Radiomics allows for detection of benign and malignant histopathology in patients with metastatic testicular germ cell tumors prior to post-chemotherapy retroperitoneal lymph node dissection. Eur. Radiol..

[22] Jing R., Wang J., Li J., Wang X., Li B., Xue F., Shao G., Xue H. (2021). A wavelet features derived radiomics nomogram for prediction of malignant and benign early-stage lung nodules. Sci. Rep..

[23] van Griethuysen J.J.M., Fedorov A., Parmar C., Hosny A., Aucoin N., Narayan V., Beets-Tan R.G.H., Fillion-Robin J.C., Pieper S., Aerts H.J.W.L. (2017). Computational Radiomics System to Decode the Radiographic Phenotype. Cancer Res..

[24] Skawran S.M., Kambakamba P., Baessler B., von Spiczak J., Kupka M., Müller P.C., Moeckli B., Linecker M., Petrowsky H., Reiner C.S. (2021). Can magnetic resonance imaging radiomics of the pancreas predict postoperative pancreatic fistula?. Eur. J. Radiol..

[25] Lin Q., Ji Y.F., Chen Y., Sun H., Yang D.D., Chen A.L., Chen T.W., Zhang X.M. (2020). Radiomics model of contrast-enhanced MRI for early prediction of acute pancreatitis severity. J. Magn. Reson. Imaging.

[26] Nougaret S., McCague C., Tibermacine H., Vargas H.A., Rizzo S., Sala E. (2021). Radiomics and radiogenomics in ovarian cancer: A literature review. Abdom. Radiol..

[27] Panico C., Avesani G., Zormpas-Petridis K., Rundo L., Nero C., Sala E. (2023). Radiomics and Radiogenomics of Ovarian Cancer: Implications for Treatment Monitoring and Clinical Management. Radiol. Clin. N. Am..

[28] Prayer F., Watzenböck M.L., Heidinger B.H., Rainer J., Schmidbauer V., Prosch H., Ulm B., Rubesova E., Prayer D., Kasprian G. (2023). Fetal MRI radiomics: Non-invasive and reproducible quantification of human lung maturity. Eur. Radiol..

[29] Xiao M.L., Fu L., Wei Y., Liu A.E., Cheng J.J., Ma F.H., Li H.M., Li Y.A., Lin Z.J., Zhang G.F. (2024). Intratumoral and peritumoral MRI radiomics nomogram for predicting parametrial invasion in patients with early-stage cervical adenocarcinoma and adenosquamous carcinoma. Eur. Radiol..

[30] Zhou Y., Zhang J., Chen J., Yang C., Gong C., Li C., Li F. (2022). Prediction using T2-weighted magnetic resonance imaging-based radiomics of residual uterine myoma regrowth after high-intensity focused ultrasound ablation. Ultrasound Obstet. Gynecol..

[31] Tellum T., Matic G.V., Dormagen J.B., Nygaard S., Viktil E., Qvigstad E., Lieng M. (2019). Diagnosing adenomyosis with MRI: A prospective study revisiting the junctional zone thickness cutoff of 12 mm as a diagnostic marker. Eur. Radiol..

[32] Harmsen M.J., Trommelen L.M., de Leeuw R.A., Tellum T., Juffermans L.J.M., Griffioen A.W., Thomassin-Naggara I., Van den Bosch T., Huirne J.A.F. (2023). Uterine junctional zone and adenomyosis: Comparison of MRI, transvaginal ultrasound and histology. Ultrasound Obstet. Gynecol..

[33] Munro M.G. (2021). Adenomyosis: A riddle, wrapped in mystery, inside an enigma. Fertil. Steril..

[34] Zheng G., Hou J., Shu Z., Peng J., Han L., Yuan Z., He X., Gong X. (2024). Prediction of neoadjuvant chemotherapy pathological complete response for breast cancer based on radiomics nomogram of intratumoral and derived tissue. BMC Med. Imaging.

[35] Dashottar S., Singh A.K., Debnath J., Muralidharan C.G., Singh R.K., Kumar S. (2015). Comparative analysis of changes in MR imaging of pre and post intrauterine progesterone implants in adenomyosis cases. Med. J. Armed Forces India.

[36] Raimondo D., Raffone A., Aru A.C., Giorgi M., Giaquinto I., Spagnolo E., Travaglino A., Galatolo F.A., Cimino M.G.C.A., Lenzi J. (2023). Application of Deep Learning Model in the Sonographic Diagnosis of Uterine Adenomyosis. Int. J. Environ. Res. Public Health.

[37] Guerriero S., Pascual M., Ajossa S., Neri M., Musa E., Graupera B., Rodriguez I., Alcazar J.L. (2021). Artificial intelligence (AI) in the detection of rectosigmoid deep endometriosis. Eur. J. Obstet. Gynecol. Reprod. Biol..

[38] Sone K., Toyohara Y., Taguchi A., Miyamoto Y., Tanikawa M., Uchino-Mori M., Iriyama T., Tsuruga T., Osuga Y. (2021). Application of artificial intelligence in gynecologic malignancies: A review. J. Obstet. Gynaecol. Res..

[39] Christiansen F., Epstein E.L., Smedberg E., Åkerlund M., Smith K., Epstein E. (2021). Ultrasound image analysis using deep neural networks for discriminating between benign and malignant ovarian tumors: Comparison with expert subjective assessment. Ultrasound Obstet. Gynecol..

[40] Taddese A.A., Tilahun B.C., Awoke T., Atnafu A., Mamuye A., Mengiste S.A. (2023). Deep-learning models for image-based gynecological cancer diagnosis: A systematic review and meta- analysis. Front. Oncol..

[41] Greener J.G., Kandathil S.M., Moffat L., Jones D.T. (2022). A guide to machine learning for biologists. Nat. Rev. Mol. Cell Biol..

[42] Martire F.G., Russo C., Selntigia A., Nocita E., Soreca G., Lazzeri L., Zupi E., Exacoustos C. (2023). Early noninvasive diagnosis of endometriosis: Dysmenorrhea and specific ultrasound findings are important indicators in young women. Fertil. Steril..

[43] Millischer A.E., Santulli P., Da Costa S., Bordonne C., Cazaubon E., Marcellin L., Chapron C. (2023). Adolescent endometriosis: Prevalence increases with age on magnetic resonance imaging scan. Fertil. Steril..

[44] Mayerhoefer M.E., Materka A., Langs G., Häggström I., Szczypiński P., Gibbs P., Cook G. (2020). Introduction to Radiomics. J. Nucl. Med..

[45] Sartoretti E., Sartoretti T., Wyss M., Reischauer C., van Smoorenburg L., Binkert C.A., Sartoretti-Schefer S., Mannil M. (2021). Amide proton transfer weighted (APTw) imaging based radiomics allows for the differentiation of gliomas from metastases. Sci. Rep..

